# *Cunninghamia lanceolata PSK* Peptide Hormone Genes Promote Primary Root Growth and Adventitious Root Formation

**DOI:** 10.3390/plants8110520

**Published:** 2019-11-18

**Authors:** Hua Wu, Renhua Zheng, Zhaodong Hao, Yan Meng, Yuhao Weng, Xiaohong Zhou, Liming Zhu, Xiangyang Hu, Guibin Wang, Jisen Shi, Jinhui Chen

**Affiliations:** 1 Key Laboratory of Forest Genetics & Biotechnology of Ministry of Education of China, Co-Innovation Center for Sustainable Forestry in Southern China, Nanjing Forestry University, Nanjing 210037, China; whua55@126.com (H.W.); haozd1992@163.com (Z.H.); mengyan194041@163.com (Y.M.); gianl13851756619@163.com (Y.W.); xiaohongzhoufei@icloud.com (X.Z.); zhulm20160918@163.com (L.Z.); jshi_1221@163.com (J.S.); 2 Fujian Academy of Forestry, Fuzhou 350012, China; zrh08@126.com; 3 Shanghai Key Laboratory of Bio-Energy Crops, School of Life Sciences, Shanghai University, Shanghai 200444, China; huxiangyang@shu.edu.cn; 4 College of Forestry, Nanjing Forestry University, Nanjing 210037, China; guibinwang99@163.com

**Keywords:** phytosulfokine, *Cunninghamia lanceolata* (Lamb.) Hook, *ClPSK* genes, root elongation, adventitious root

## Abstract

Phytosulfokine-α (PSK-α) is a newly discovered short peptide that acts as a phytohormone in various plants. Previous studies have shown that PSK-α is critical for many biological processes in plants, such as cell division and differentiation, somatic embryogenesis, pollen germination and plant resistance. In this study, we cloned two PSK homolog genes from *Cunninghamia lanceolata* (Lamb.) Hook (Chinese fir), *ClPSK1* and *ClPSK2*, and characterized their function in root development. Quantitative RT-PCR analyses showed that both *ClPSK1* and *ClPSK2* were expressed in vegetative organs, mainly in roots. Transgenic *Arabidopsis* plants overexpressing *ClPSK1* or *ClPSK2* showed a higher frequency of adventitious root formation and increased root length. The expression of genes in *Arabidopsis* that are involved in stem cell activity (*PLT1*, *PLT2* and *WOX5*), radial organization of the root (*SHR* and *SCR*) and cell cycle (*CYCB1;1*, *CYCD4;1*, *CDKB1;1* and *RBR*) were significantly up-regulated, which may contribute to the elongation of the primary root and the formation of adventitious root in transgenic lines. Our results suggest that *ClPSKs* play an important role during root growth and development.

## 1. Introduction

*Cunninghamia lanceolata* (Lamb.) Hook (Chinese fir) is an important native evergreen tree in southern China. Because of its elite wood attributes and high timber productivity, Chinese fir occupies a prominent role regarding ecological and commercial prospects.

In natural environments, the growth and development of plants are strongly influenced by surrounding conditions. This plasticity allows plants to adapt to a changing and adverse environment, thus compensating for their immobile lifestyle. In many cases, the plant’s response to developmental conditions is mediated by non-proteinaceous phytohormones, such as auxin, cytokinin, gibberellin, abscisic acid, ethylene, etc. 

In addition, many studies have shown that peptides are similarly important in plant signaling [1]. Peptides are generally defined as polypeptide chains smaller than 100 amino acid residues. Phytosulfokine-α (PSK-α) belongs to a group of sulfated plant peptides [2] and is derived from a precursor polypeptide that is 80–120 amino acids in length; such prepropeptides are often part of small gene families [3,4]. YIYTQ is a highly conserved pentapeptide sequence found in PSK homologs, that is located in the C-terminus of the precursor peptide [4]. Many studies have shown that PSK can promote cell division and differentiation, promote somatic embryogenesis, regulate pollen germination and improve plant resistance [3,5,6]. Although PSK-α has been identified in diverse plant species [6,7,8], the role of PSK-α in Chinese fir has not been reported.

There are six *PSK* genes in *Arabidopsis*, but five expressed *PSK* genes exist that contain the canonical YIYTQ sequence [1]. *AtPSK1* and *AtPSK3* were both expressed in root tips, and *AtPSK2*, *AtPSK4* and *AtPSK5* displayed strong overlapping activities in the central cylinder of the differentiated part of main and lateral roots [9]. *Arabidopsis* has two PSK receptor genes, *AtPSKR1* and *AtPSKR2. AtPSKR1* was expressed in shoots and roots at seemingly low levels, and *PSK* signaling through *AtPSKR1* promotes cell elongation [9,10,11]. *AtPSKR2* is expressed in the root tip and affects cell proliferation [1,9]. Taken together, PSK precursor and receptor genes might thus initiate divergent signaling pathways, leading to the activation of different cellular processes, both of which contribute to enhanced root growth. Low concentrations of brassinosteroid (BR) epi-brassinolide promote root elongation growth [12]. PSK signaling in the epidermis is dependent on the presence of BR signaling and BR synthesis and perception are a prerequisite for the PSK signaling of cell elongation [13]. These results indicated that PSK signaling of root growth requires brassinosteroid synthesis and may thus act as a mobile signal in the PSK response. ERF115 transcription factor as a rate-limiting factor of quiescent center (QC) cell division, acting as a transcriptional activator of the phytosulfokine PSK5 peptide hormone, whereas QC proliferation is driven by brassinosteroid-dependent ERF115 expression [14]. This evidence suggested that the PSK and brassinosteroid signal pathways interact in elongating cells and the QC.

The formation of adventitious roots, a dynamic phenomenon, involves the regeneration of cells and organs. Previous studies have shown that many compounds have been reported to promote adventitious root formation, including auxin [15,16,17,18] and abscisic acid [19,20]. PSK-α increases the formation of adventitious roots by promoting plant organogenesis in cucumber hypocotyls, acting possibly via enhancing plant cell proliferation [21]. Various genes involved in adventitious root formation have been studied [18,22,23,24]. The cell cycle marker genes, such as, *CYCB1;1*, *CYCD4;1*, *CDKB1;1* and *RETINOBLASTOMA-RELATED* (*RBR*) are known to be important for regulating meristematic activity in roots [25,26,27,28,29,30]. AP2 family transcription factors, such as *PLETHORA1* (*PLT1*), *PLETHORA2* (*PLT2*), GRAS family transcription factors like *SHORTROOT* (*SHR*), *SCARERROW* (*SCR*), as well as the *WUSCHEL RELATED HOMEOBOX* transcription factor *WOX5* have been extensively studied and found to be essential for primary root growth and development [18,30,31,32,33]. *PLT* is necessary for root formation; it acts dose dependently, with high PLT levels maintaining stem cells [34,35]. *SHR* and *SCR* mainly regulate asymmetric division and QC maintenance of endothelial and cortical blast cells [36,37]. *WOX5* is expressed at the initiation of the lateral root, specifically expressed in the QC [38] and involved in regulating cell division of the root apical meristem (RAM) [39]. *ERF115* is an AP2-type transcription factor gene that is expressed in the QC. ERF115-induced QC cell divisions depend on PSK, indicating that PSK signaling occurs in the QC [14]. These findings suggest that *PSK* and *WOX5* signaling pathways interact in the QC.

We aimed to study whether in Chinese fir, PSK signaling similarly contributes to root development and could therefore aid in the plant’s adaptability to changing environmental conditions. Therefore, we cloned and characterized two putative Chinese fir *PSK* genes. Using cross-species transgenesis studies in *Arabidopsis thaliana*, we found that the rate and frequency of adventitious root formation was increased by over-expression of *ClPSK* corresponding to an increase in the expression levels of *PLT1*, *PLT2*, *SHR*, *SCR* and *WOX5*. These results indicate that *ClPSK* may play an important role in adventitious root formation. We demonstrate that overexpression of *ClPSK* improves root growth and promotes formation of adventitious root in *Arabidopsis thaliana*. These findings may aid future studies aimed at improving Chinese fir adaptability.

## 2. Results

### 2.1. Molecular Cloning and Characterization of the ClPSK Genes

**Figure 1:**
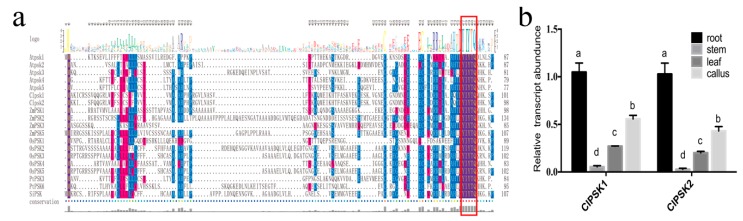
*ClPSK* is predicted to encode a phytosulfokine precursor protein. (**a**) Amino acid multiple sequence alignment between ClPSK and phytosulfokine-a (PSKs) from other species. The highly conserved single PSK domain (YIYTQ) is shown with a red rectangular box. The alignment was built from the following amino acid sequences: AtPSK1 (NP_172816); AtPSK2 (NP_179871); AtPSK3 (NP_566871); AtPSK (NP_566926); AtPSK5 (NP_201388); ZmPSK1 (NP_001105796); ZmPSK2 (NP_001150909); ZmPSK3 (NP_001147408); ZmPSK5 (NP_001146993); OsPSK1 (NP_001158130); OsPSK2 (NP_001065756); OsPSK3 (NP_001066155); OsPSK4(NP_001058803); OsPSK5 (NP_001066155); PtPSK3 (EEE90779); PtPSK6 (ABK92977); SiPSK (KQK99873). At: *Arabidopsis thaliana*; Cl: *Cunninghamia lanceolata*; Zm: *Zea mays*; Os: *Oryza sativa*; Pt: *Populus trichocarpa*; Si: *Setaria italica*. (**b**) Relative expression levels of *ClPSK1/2* in the root, stem, leaf and embryonic callus of Chinese fir as determined by quantitative real-time PCR analysis. Error bars represent the standard deviations of three independent biological replicates. Different letters indicate significant differences between treatments, significant differences in mRNA levels were detected by the ANOVA test (*P* < 0.05).

We cloned the *ClPSK1* and *ClPSK2* sequences from Chinese fir embryogenic callus. The cDNA of *ClPSK1* is 303 bp long and the open reading frame encodes 100 amino acids. The cDNA of *ClPSK2* is 294 bp long, encoding 97 amino acids. PSK has a predicted secretion signal at the N-terminus and a highly conserved single PSK domain (YIYTQ) close to the C-terminus (Figure 1a). Sequence alignment reveals that the PSK domain is identical to that of other known *PSK* precursor amino acid sequences (Figure 1a). However, the remaining sequence is highly divergent across species.

To investigate the spatial distribution of *ClPSK* transcripts, quantitative real-time PCR (qRT-PCR) analysis was performed in different tissues of Chinese fir. This analysis indicated that *ClPSK* was expressed in the root, stem, leaf and embryonic callus of Chinese fir, and that the relative expression of *ClPSK1* and *ClPSK2* genes was highest in roots ((Figure 1b).

### 2.2. Overexpression of ClPSK Promotes Root Growth in Arabidopsis Thaliana

**Figure 2:**
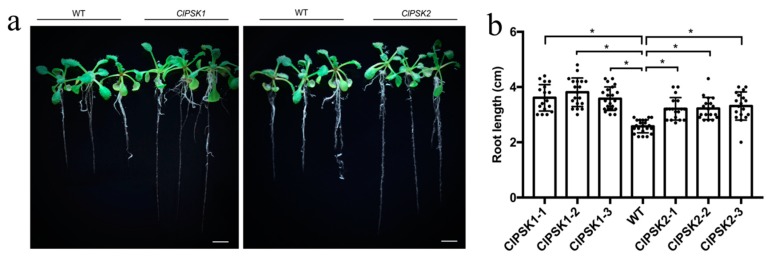
Overexpression of *ClPSK* promotes root growth in *Arabidopsis thaliana* plants. (**a**) Overexpression of *ClPSK* genes increases *Arabidopsis* root length. Representative photographs of *Arabidopsis* wild type (WT), *ClPSK1* and *ClPSK2* plants, grown for 15 d on plates. Bar = 0.5 cm. (**b**) Measurement of root length in *35S::ClPSK* and wild type *Arabidopsis*. The average root length per plant was determined for *Arabidopsis* wild type (WT), three independent transgenic *ClPSK1* lines (ClPSK1-1, ClPSK1-2, ClPSK1-3) and three independent transgenic *ClPSK2* lines (ClPSK2-1, ClPSK2-2, ClPSK2-3). Plants were grown for 15 d on plates. Black points represent individual data points. The center line represents the mean, and error bars represent standard error (SE). *n* ≥ 20 biological replicates. Asterisks indicate statistically significant difference between WT and transgenic lines, as determined by Student’s *t*-test (*P* < 0.05).

To determine functionality of *ClPSK1* and *ClPSK2*, we overexpressed *ClPSK* genes under the control of the CaMV 35S promoter in *Arabidopsis thaliana*. We found that the roots of transgenic plants were significantly longer than those of the wild type (Figure 2a). This effect was consistent across three independent transgenic lines for each gene, with root lengths increased by 39.4%, 50.2% and 38.5% in the transgenic lines ClPSK1-1, ClPSK1-2 and ClPSK1-3, respectively, and increased by 23–28% in lines ClPSK2-1, ClPSK2-2 and ClPSK2-3, compared to the wild type (Figure 2b). These results demonstrate that overexpressing *ClPSK* in *Arabidopsis thaliana* promotes primary root growth.

### 2.3. Overexpression of ClPSK Gene Promotes Organ Differentiation

**Figure 3:**
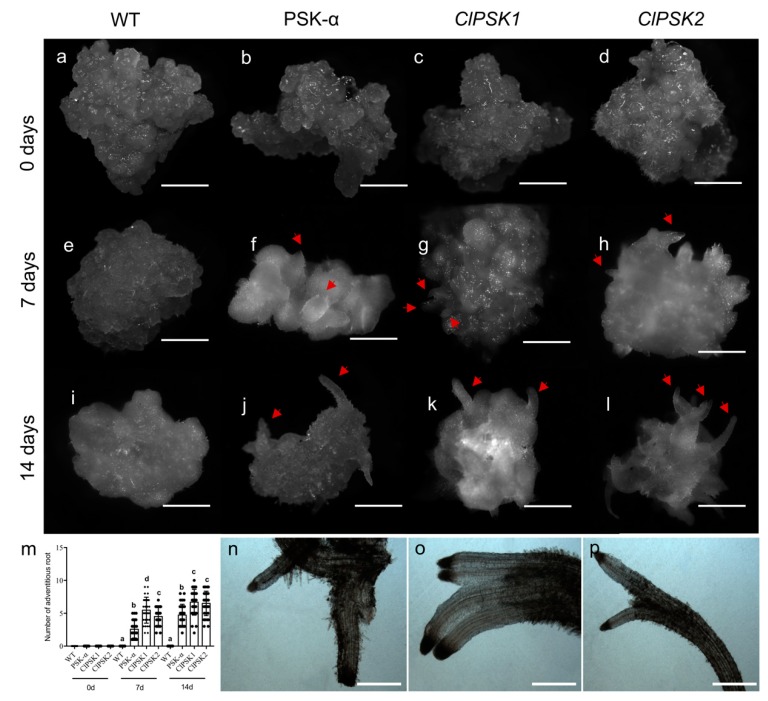
Overexpression of the *ClPSK* gene promotes organ differentiation. (**a**–**l**) Overexpression of *ClPSK* genes promotes organogenesis in *Arabidopsis*. Representative micrographs of wild-type, PSK-α treated and *35S::ClPSK1/2 Arabidopsis* callus at 0 (**a**–**d**), 7 (**e**–**h**) and 14 (**i**–**l**) days of culture in a basic Murashige and Skoog (MS) liquid medium. Transparent protrusions (indicated by red arrows) occurred on the surface of PSK-α-treated wild-type callus (**f**) and transgenic callus i.e., *ClPSK1* (**g**) and *ClPSK2* (**h**) at 7 days. Elongated transparent protrusions on the surface of the PSK-α-treated wild-type callus (**j**) and transgenic callus *ClPSK1* (**k**) and *ClPSK2* (**l**) at 14 days. Bar = 2 mm. (**m**) The number of adventitious roots on the surface of wild-type (WT), PSK-α-treated wild-type (PSK-α) and transgenic callus (ClPSK1 and ClPSK2) at 0, 7 and 14 days of culture. Black points represent individual data points. The center line represents the mean, and error bars represent standard error (SE), *n* = 30 biological replicates. Different letters indicate statistically significant differences from WT (*P* < 0.05) were obtained using an ANOVA test. (**n**–**p**) Adventitious roots formed on *Arabidopsis* callus. Representative micrographs obtained using an inverted microscope. PSK-α-treated wild-type callus (**n**) and transgenic callus *ClPSK1* (**o**) and *ClPSK2* (**p**) at 14 days. Bar = 0.5 mm.

Previous studies have shown that PSK-α can promote organogenesis in plants [21]. To explore the function of *ClPSK*, we observed organogenesis from *35S::ClPSK* and wild-type callus treated with basic liquid Murashige and Skoog (MS) medium. We found no apparent changes in wild-type callus after liquid suspension culture for 7 days, while transparent protrusions could be observed on the surface of *35S::ClPSK* callus (Figure 3a–l). In addition, we observed transparent protrusions from wild-type callus grown in basic liquid MS medium supplemented with 0.1 mg L^−1^ PSK-α after 7 days of liquid culture (Figure 3a–l). After 14 days, the transparent protrusions became significantly longer (Figure 3m). Observed through the microscope, we found the transparent protrusions formed on the callus surface to display obvious root characteristics (Figure 3n–p). These results demonstrate that *ClPSK* can induce organogenesis and lead to the production of adventitious roots on callus surface in *Arabidopsis thaliana*.

### 2.4. Overexpression of ClPSK Genes Upregulates the Expression of Genes Related to Root Morphogenesis

**Figure 4:**
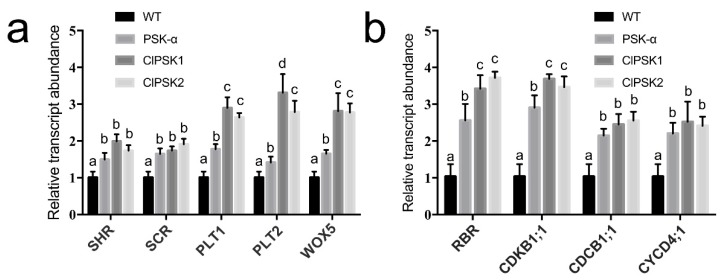
Overexpression of *ClPSK* genes upregulate the expression of genes related to root morphogenesis. (**a**) *ClPSK1/2* induce expression of root patterning genes in *Arabidopsis* callus. Relative expression levels of genes associated with root development in wild-type (WT), PSK-α treated (PSK-α) and *35S::ClPSK1/2* (ClPSK1 and ClPSK2) callus grown 7 days in a basic MS liquid medium. Both *ClPSK1/2* overexpression and exogenous treatment upregulate expression of *SHR*, *SCR*, *PLT1*, *PLT2* and *WOX5*. Different letters indicate statistically significant differences between treatments in one genotype. Relative expression data are expressed as the mean ± SD, *n* = 3 biological replicates, and statistically significant differences from WT (*P* < 0.05) were obtained using an ANOVA test. (**b**) *ClPSK1/2* induce expression of cell cycle genes in *Arabidopsis* callus. Relative expression levels of cell-cycle genes in wild-type (WT), PSK-α treated (PSK-α) and *35S::ClPSK1/2* (ClPSK1 and ClPSK2) callus grown 7 days in basic MS liquid medium. Both *ClPSK1/2* overexpression and exogenous treatment upregulate expression of *RBR, CDKB1;1, CYCB1;1* and *CYCD4;1*. Different letters indicate statistically significant differences between treatments in one genotype. Relative expression data are plotted as the mean ± SD, *n* = 3 biological replicates, and statistically significant differences from WT (*P* < 0.05) were obtained using an ANOVA test.

To further investigate the relationship between *ClPSK1/2* and root formation, we analyzed the expression of genes associated with root development, such as *PLT1*, *PLT2*, *SHR*, *SCR* and *WOX5* in *Arabidopsis* callus. We found that these genes were up-regulated in callus overexpressing *ClPSK* genes or treated with PSK-α cultured for 7 days in suspension culture, with *ClPSK* overexpression being more effective at inducing some of these genes compared to PSK-α treatment (Figure 4a). These data support at a molecular level that overexpression of *ClPSK* promotes root morphogenesis.

Root meristem activity is required to accelerate the rate of cell division for a continuous supply of new cells. Acceleration of cell elongation contributes to enhanced root growth, but it is not sufficient to promote long-term growth without the production of new cells from the meristem. Therefore, we tested the expression level of genes involved in cell division [25,26,27,28,29,30]. The expression levels of *CYCB1;1, CYCD4;1, CDKB1;1* and *RBR* are up-regulated in transgenic lines and wild-type treated with 0.1mg L^−1^ PSK-α, which were measured after callus was cultured for 7 days in suspension culture (Figure 4b). Moreover, the expression levels of *CDKB1;1* and *RBR* of transgenic lines were slightly higher than PSK-α-treated wild-type (Figure 4b). These results indicate that PSK is able to promote cell division.

## 3. Discussion

Here, we describe the molecular cloning and characterization of two *ClPSK* gene homologs originating from Chinese fir, that encode the precursor of PSK-α. We detected high expression of both *ClPSK1* and *ClPSK2* genes in Chinese fir root (Figure 1b). Previous studies have shown that the primary root of plants overexpressing *AtPSK4* is significantly longer than in the wild type by promoting root growth by enhancing cell elongation [9,40], and that *GhPSK* can promote the elongation of roots in *Arabidopsis thaliana* [41]. In this study, our results show that overexpression of *ClPSK1* or *ClPSK2* can significantly promote root growth in *Arabidopsis thaliana*, consistent with the phenotypes of *AtPSK4* and *GhPSK* overexpression in *Arabidopsis thaliana*.

The growth and development of plant roots involves complex regulatory networks, as well as key transcription factors, such as *PLT1*, *PLT2*, *SHR*, *SCR* and *WOX5*, which have been shown to be involved in root formation. We observed that *ClPSK1/2* overexpression or PSK-α treatment induced adventitious roots on the callus surface after 14 days. Correlating with these findings, increased levels of *ClPSK1/2* enhanced the expression levels of *PLT1*, *PLT2*, *SHR*, *SCR* and *WOX5* (Figure 4a). Taken together, these data suggest that *ClPSK1* and *ClPSK2* effect gene networks related to root development, and that they have functions involved in root morphogenesis. The positive contribution of PSK signaling to promoting adventitious root formation from callus has not been reported previously, although PSK has been implicated in inducing adventitious root formation from cucumber hypocotyls and adventitious bud formation from callus of *Antirrhinum majus* [21,42].

In addition, we found that expression of the cell cycle marker genes (*CYCB1;1*, *CYCD4;1*, *CDKB1;1* and *RBR*) was up-regulated in the adventitious roots *of 35S::ClPSK1/2* transgenic lines (Figure 4b). *CYCD4;1* is abundantly expressed during the initiation of the root primordium, and *CYCD4;1* transcripts accumulate in the vascular tissue of roots, as well as in lateral root primordia [29]. Overexpression of *CYCB1;1* can promote root meristem division and increases root elongation [35]. CDKB is a type of CDK kinase that acts in the S and G2/M phases of the cell cycle [43,44]. Inhibition of CDKB activity can cause serious defects in plant meristems [45]. The expression of *RBR* directly affects the number and characteristics of cells in the root apical meristem (RAM) in *Arabidopsis thaliana*. Furthermore, *RBR* is required for stem cell maintenance, cell differentiation and lateral organ production [46]. These genes are all involved in the cell-cycle, but how does PSK impinge on the molecular control of the cell cycle? Hormone signaling is a key component of root growth, as a plant’s response to changing environmental or developmental conditions are mediated by hormones. PSK is a new plant hormone, but its physiological characteristics and its mechanism of action are still unclear. Our study demonstrates that *ClPSK* gene promotes organ differentiation, and it highlights a novel plant growth regulator that may be used for organogenesis and somatic embryogenesis of Chinese fir. Our findings provide a preliminary understanding of *ClPSK* function, and give an important basis for further understanding the *PSK* signal transduction mechanism and its role in plant growth and development.

## 4. Materials and Methods

### 4.1. Plant Materials and Culture Conditions

Experiments were performed with *Arabidopsis thaliana* ecotype Columbia (Col-0). Arabidopsis seeds were surface-sterilized with 75% (v/v) ethanol for 30 s, treated with 0.1% (w/v) HgCl_2_ for 2.5 min, germinated on Murashige and Skoog (MS) medium, and cultured at 22 ℃ and 70% humidity with a 16/8 h light/dark cycle. For the measurement of root length of T3 homozygous plants were measured after growing for 15 day after germination.

For callus induction, *Arabidopsis thaliana* seeds germinated on MS medium for 2 weeks, then *Arabidopsis* leaves were transferred to MS solid medium containing 2 mg L^−1^ 2,4-dichlorophenoxyacetic acid, 0.2 mg L^−1^, benzyl-aminopurine, 500 mg L^−1^ casein hydrolysate (CH) (Sigma, Darmstadt, Germany) and 30 g L^−1^ sucrose, after which they were cultured at 23 ℃ in darkness. Callus was transferred to basic MS liquid medium consisting 30 g L^−1^ sucrose, and cultured at 23 ℃ in darkness so as to induce the formation of adventitious roots.

Roots, stems and leaves of Chinese fir seedlings germinated from somatic embryos and embryogenic callus were induced following the methods previously described by Zhou Xiaohong [47].

### 4.2. Gene Cloning and Construction of Vectors and ClPSK Overexpression Lines

Based on transcriptome data (shared in the Lab) of Chinese fir, the full-length cDNA clones of two *ClPSK* genes, *ClPSK1* and *ClPSK2*, were cloned from embryogenic callus of Chinese fir and used the NCBI ORF finder to detect open reading frames (https://www.ncbi.nlm.nih.gov/orffinder/). Briefly, *ClPSK1* and *ClPSK2*, were cloned and inserted into the pBI121 vector with restriction enzymes *Bam* HI and *Xba* I (NEB) to generate the overexpression vector 35S::PSK. *Arabidopsis thaliana* plants were transformed via the floral dipping method using *Agrobacterium tumefaciens* [48]. Resistant plants were selected using 50 mg L^−1^ kanamycin. The transgenic plants were selected by the PCR analysis. The single-locus homozygous transgenic lines were then identified by the genetic analyses of segregation at 3:1 in the T1 generation and no separations in the T2 and T3 generations (*n* > 30). All primers used for gene cloning were listed in Table S1.

### 4.3. Characterization of the ClPSK Genes Analysis

The amino acid multiple sequence alignment between ClPSK and phytosulfokine-a (PSKs) from other species was performed using TEXshade (https://ctan.org/pkg/texshade). The PSK protein sequences of other species were downloaded from the National Center for Biotechnology Information (NCBI) web site (http://www.ncbi.nlm.nih.gov).

### 4.4. Quantitative Real-Time PCR

Total RNA was isolated using the RNAprep Pure Plant Kit (Tiangen, Beijing, China) and then was reversely transcribed to cDNA with a reverse transcriptase kit (Roche, Shanghai, China). Quantitative real-time PCR was performed using the LightCycler 480 System (Roche Applied Science, Shanghai, China), as previously described [47]. The *CleIF-3* housekeeping gene was selected as the endogenous reference gene for the qRT-PCR analysis of cambial development in Chinese fir [49], and primers for qRT-PCR are listed in the Table S2. All primers used for qRT-PCR of *Arabidopsis thaliana* were listed in Table S3. Each measurement was performed using three biological samples and each test of sample was conducted with three replicates. Relative gene expression was performed using the 2^−∆∆CT^ method [50].

### 4.5. Morphological Analysis

The morphology and characteristics of adventitious roots was evaluated using a stereoscope (Leica, S8AP0) and micrographs were obtained using an inverted microscope (Leica, DMI4000, Wetzlar, Germany).

## Supplementary Materials

The following are available online at https://www.mdpi.com/2223-7747/8/11/520/s1, Table S1: Primers were used for gene cloning, Table S2: Primer sets used for quantitative qRT-PCR in Chinese fir, Table S3: Primer sets used for quantitative qRT-PCR in *Arabidopsis thaliana*.
