# Supplementary material for: Cunninghamia lanceolata PSK Peptide Hormone Genes Promote Primary Root Growth and Adventitious Root Formation
Source: Plants (Basel). 2019 Nov 18;8(11):520. doi: 10.3390/plants8110520 (PMC6918316; doi:10.3390/plants8110520)
Supplement: Supplementary file 1 [file plants-08-00520-s001.pdf]

Supplementary Material

Supplementary Tables

Supplementary Table S1. Primers were used for gene cloning.

Primer name	Primer sequence (5′–3′)
ClPSK1	F: ATGGCGAAAATCTGTAGAAGTTCAGTG<br>R: TGGGCTCTTGTGTTGTTGTGTATAGATG
ClPSK2	F: ATGAAAAAAATTTCATTTCAACAGGGAAG<br>R: TGGGTTCTTGTGGTGTTGTGTATAAATG

Supplementary Table S2. Primer sets used for quantitative qRT-PCR in Chinese fir.

Primer name	Primer sequence (5′–3′)
PSK1	F: ATGGCGAAAATCTGTAGAAGTTCAG<br>R: GCATTCTTCTTCATTCATATCATTG
PSK2	F: ATGAAAAAAATTTCATTTCAACAGG<br>R: TATCATTGCCACATTCATTCCCTAC
eIF-3	F: TCTGCATCCCAATGATTTGT<br>R: TCTTCAGATGTTGCTTGCCT

Supplementary Table S3. Primer sets used for quantitative qRT-PCR in *Ar*abidopsis *thaliana*.

Primer name	Primer sequence (5′–3′)
AtActin	F: GGCTCCTCTTAACCCAAAGGC<br>R: CACACCATCACCAGA ATCCAGC
AtCYCD4;1	F: GATGAGGGCATGATGTTGACG<br>R: CCAAACTGGTGTACTTCACAAGC
AtCYCB1;1	F: CTCAAAATCCCACGCTTCTTGTGG<br>R: CACGTCTACTACCTTTGGTTTCCC
AtCDKB1;1	F: GGTGGTGACATGTGGTCTGTTGG<br>R: CGCAGTGTGGAAACACCCGG
AtRBR	F: CTCATAAGTCGCCTGCTGCTAAG<br>R: TTGCTGTGCTCACTGGTGTTG
AtSCR	F: TGGTCGAGGAGGAGAGGAATAG<br>R: CGCTTGTGTAGCTGCATTTCC
AtSHR	F: ACACTGTACCATCGACCAAACACC<br>R: TAGCGGTTGGAGGACCATCG

AtPLT1	F: ACGAAAACCAATCCAACCAC
	R: CCTAGACTGGCCTTCCCTTC
AtPLT2	F: GTTACCTACAGTCGTCACTTGTGC
	R: ACTCTTGTCTCGTCATGTTTTTCA
AtWOX5	F: AAGCTTGCGAAGAAGATTGTCAAGAGG
	R: GATATCCGTGGTGGTCTCTCGAATATA
